# Predicting cross-tissue hormone–gene relations using balanced word embeddings

**DOI:** 10.1093/bioinformatics/btac578

**Published:** 2022-08-24

**Authors:** Aditya Jadhav, Tarun Kumar, Mohit Raghavendra, Tamizhini Loganathan, Manikandan Narayanan

**Affiliations:** Department of Computer Science and Engineering, Indian Institute of Technology (IIT) Madras, Chennai, India; Department of Computer Science and Engineering, Indian Institute of Technology (IIT) Madras, Chennai, India; Initiative for Biological Systems Engineering, IIT Madras, Chennai, India; Robert Bosch Centre for Data Science and Artificial Intelligence, IIT Madras, Chennai, India; Department of Information Technology, National Institute of Technology Karnataka, Surathkal, India; Initiative for Biological Systems Engineering, IIT Madras, Chennai, India; Robert Bosch Centre for Data Science and Artificial Intelligence, IIT Madras, Chennai, India; Department of Computer Science and Engineering, Indian Institute of Technology (IIT) Madras, Chennai, India; Initiative for Biological Systems Engineering, IIT Madras, Chennai, India; Robert Bosch Centre for Data Science and Artificial Intelligence, IIT Madras, Chennai, India

## Abstract

**Motivation:**

Inter-organ/inter-tissue communication is central to multi-cellular organisms including humans, and mapping inter-tissue interactions can advance system-level whole-body modeling efforts. Large volumes of biomedical literature have fostered studies that map within-tissue or tissue-agnostic interactions, but literature-mining studies that infer inter-tissue relations, such as between hormones and genes are solely missing.

**Results:**

We present a first study to predict from biomedical literature the hormone–gene associations mediating inter-tissue signaling in the human body. Our BioEmbedS* models use neural network-based Biomedical word Embeddings with a Support Vector Machine classifier to predict if a hormone–gene pair is associated or not, and whether an associated gene is involved in the hormone’s production or response. Model training relies on our unified dataset Hormone-Gene version 1 of ground-truth associations between genes and endocrine hormones, which we compiled and carefully balanced in the embedded space to handle data disparities, such as between poorly- versus well-studied hormones. Our BioEmbedS model recapitulates known gene mediators of tissue–tissue signaling with 70.4% accuracy; predicts novel inter-tissue communication genes in humans, which are enriched for hormone-related disorders; and generalizes well to mouse, thereby holding promise for its extension to other multi-cellular organisms as well.

**Availability and implementation:**

Freely available at https://cross-tissue-signaling.herokuapp.com are our model predictions & datasets; https://github.com/BIRDSgroup/BioEmbedS has all relevant code.

**Supplementary information:**

Supplementary data are available at *Bioinformatics* online.

## 1 Introduction

Inter-tissue communication forms the basis for life and health in multi-cellular organisms, and multiple organs/tissues are often affected in a systemic fashion in complex diseases [e.g. type 2 diabetes (Priest and Tontonoz, 2019) or cancer cachexia (Argilés *et al.*, 2018)]. A grand goal of systems biology is to develop whole-body systemic models of healthy/disease conditions; and that calls for mapping not only the within-tissue biomolecular interactions, but also the crucial across-tissue interactions (Castillo-Armengol *et al.*, 2019). Within-tissue interactions are focused heavily in current genomic or literature-mining studies—for instance, several recent methods that examine single-cell/spatial omics data to predict ligand-receptor-based cell–cell interactions (Armingol *et al.*, 2021; Türei *et al.*, 2021) have focused largely on proximal inter-cell communication within a single tissue/organ, and not on inter-tissue interactions that are distal or endocrine in nature. Recently developed whole-body *metabolic* models for humans (Brul and Angione, 2019), such as the Harvey and Harvetta models [encompassing 26+ organs and their metabolic interactions culled from literature and refined by genomic or other experimental data (Thiele *et al.*, 2020)] are promising, but models of similar scale at the *gene-level* are lacking.

Emerging multi-tissue genomic datasets [e.g. the Genotype-Tissue Expression or GTEx (The GTEx Consortium, 2020) and Human Cell Atlas (Regev *et al.*, 2017) data] have encouraged some gene-level efforts to address the dearth of studies on inter-tissue signaling, and there is an urgent need to augment/validate such data-driven whole-body gene networks by literature-driven approaches. An ideal literature-mining system would extract relations of the form ‘Gene X in Tissue A interacts with Gene Y in Tissue B via a mediating signaling molecule’ from a literature corpus, and importantly also predict new relations using the semantic/syntactic attributes of the relevant genes and signaling molecules mentioned in the corpus.

In this work, we harness the large volume of biomedical literature [present in repositories like PubMed (Westergaard *et al.*, 2018) with ∼23 million abstracts] to identify genes involved in inter-tissue signaling mediated by hormones. We specifically focus on endocrine hormones, as they are a popular class of signaling molecules and endocrine biology has revealed and continues to reveal many hormones and their regulating genes. For example, in the pancreas tissue, gene *INS* produces the well-studied insulin hormone, which is processed and secreted into the blood with the help of other gene products and biomolecules; and the protein encoded by the *INSR* gene is the primary receptor of the hormone in muscle and other tissues, where INSR protein gets activated by the insulin hormone and affects several other genes downstream to regulate glucose uptake by body cells. A more recent example is a new type of vesicle-mediated communication between liver and adipose tissues involving non-protein-coding (microRNA) genes (Chen and Pfeifer, 2017; Zhao *et al.*, 2020). The goal of this work is to extract all such hormone–gene relations, and classifying the hormone-associated genes as either source (genes aiding in hormone production/processing/secretion at a source tissue) or target (genes responding to the hormone directly via binding or as a downstream response at a target tissue).

Several challenges stand in the way of our goal of extracting hormone–gene relations from literature, including:

1. Lack of a unified database of ground-truth hormone–gene associations, since current hormone databases like HMRbase (Rashid *et al.*, 2009) and EndoNet (Dönitz and Wingender, 2014) focus on the primary gene coding for a (peptide) hormone and the primary receptor genes, and not on the many other genes involved in hormonal processing/response.
2. Severe imbalance in known hormone–gene relations both in the number of source versus target genes of each hormone, and of associated genes per hormone, which varies widely across hormones (e.g. insulin is better studied than many other hormones).
3. Lack of standard *in silico* strategies for large-scale validation of novel hormone–gene predictions using independent data on inter-tissue signaling.

Our work attempts to address the barriers above to characterize inter-tissue signaling, and current studies have addressed only the second challenge above and that too in very different contexts/applications. Specifically, current literature-mining studies largely extracted relations among entities that are tissue-agnostic (i.e. does not depend on the tissue) or within-tissue (i.e. happens within one or more of the tissues or cell types), and includes relations, such as disease–gene (Bhasuran and Natarajan, 2018), gene–phenotype (Xing *et al.*, 2018), drug–drug (Yan *et al.*, 2013), or protein–protein/gene–gene (Szklarczyk *et al.*, 2015; Yu *et al.*, 2018) types, or subsets of them (Bravo *et al.*, 2015; Junge and Jensen, 2020). Besides using traditional Natural Language Processing (NLP) features based on syntax/semantics, these methods also exploit modern advances like word embeddings, which are vector representations of words that are learnt via deep learning models [Word2Vec (Mikolov *et al.*, 2013) or FastText (Bojanowski *et al.*, 2017) or BioBERT (Lee *et al.*, 2020)] to capture the semantic similarities and relationships among words. Examples include a joint ensemble learning approach by Bhasuran and Natarajan (2018) for disease–gene predictions, which builds upon a Support Vector Machine (SVM) classifier called BeFree introduced earlier by Bravo *et al.* (2015); and an approach by Park *et al.* (2019) that combines traditional NLP techniques with Word2Vec embeddings.

Given this context, our work on systematic prediction of genes mediating inter-tissue signaling makes three main contributions:

1. Our work is the first study to predict hormone–gene associations from biomedical literature, with our focus on inter-tissue communication setting it apart from earlier literature-mining studies on predicting tissue-agnostic or within-tissue interactions.
2. Our work is enabled by expressly compiling a database of ground-truth hormone–gene associations called Hormone-Gene version 1 (HGv1), and balancing it in the space of mapped word embeddings to avoid well-studied hormones and source-versus-target genes’ imbalance from unduly influencing our model predictions.
3. Our models BioEmbedS (Biomedical Word Embeddings + SVM) and BioEmbedS-TS (Biomedical Word Embeddings + SVM—Target versus Source), which are SVM classifiers trained on these balanced word embeddings, not only corroborate existing hormone–gene links and hormone source versus target genes (collated in HGv1, respectively at an average accuracy of 70.4% and 79%), but also predict novel gene associations of hormones. These novel genes are enriched for diseases known to be related to the corresponding hormone, across many different hormones.

These contributions of our study, concretely demonstrated in the human and generalized to the mouse organism, also bode well for future works on other multi-cellular organisms. The set of hormone–gene predictions from our study has varied applications from accelerating literature curation efforts around whole-body modeling to prioritizing new experiments to dissect inter-organ communication. For these applications, our model accuracies as reported above are adequate, since we take our hormone–gene predictions as prioritized hypotheses that are then validated via manual literature curation or molecular biology experiments. Substantial time and cost savings can result from employing a prioritized list of curation or experimental tasks to build a whole-body gene network model of normal physiology or various hormone-related disorders, since without prioritization, the huge space of all pairwise interactions among thousands of genes in tens of organs of interest would need to be considered.

## 2 Results

### 2.1 Overview of our BioEmbedS* models and HGv1 ground-truth dataset

**Fig. 1.**
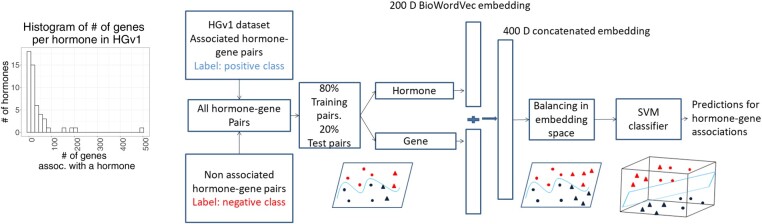
BioEmbedS model overview: our BioEmbedS model predicts if a hormone–gene pair is associated or not from D-dimensional word embedding vectors of the hormone name and the gene symbol. Our HGv1 dataset is crucial for systematic training/evaluation of our model, after its proper balancing to handle variability in available information for different hormones (see inset histogram; ‘assoc.’ stands for associated). In the toy-example shown, circles and triangles indicate hormone–gene pairs for two illustrative hormones; and below-the-boundary blue and above-the-boundary red symbols, respectively, denote the positive (associated) and negative (non-associated) genes for each hormone. The positive/negative classes are balanced across the two hormones, before separating them in a higher dimensional space using a SVM classifier. BioEmbedS-TS model has source and target genes for a hormone in place of positive and negative genes (A color version of this figure appears in the online version of this article.)

We develop two classification models—BioEmbedS to predict hormone–gene associations, and BioEmbedS-TS to classify an associated gene into source versus target set of a hormone. These models, referred jointly as BioEmbedS*, are SVM classifiers trained and evaluated using our ground-truth dataset HGv1, and use word embeddings as input features (Fig. 1). An embedding of a word, such as a hormone name or gene symbol, is a vector representation of the word that is learnt via a deep learning model to capture the context of this word in a corpus. By using word embeddings, specifically the 200-dimensional vectors called BioWordVec (Zhang *et al.*, 2019) derived from the PubMed corpus, we not only capitalize on this modern advance in literature mining, but also importantly balance the skew in our HGv1 dataset using established balancing techniques that can work only with feature vectors and not direct text (see Section 4 for details). The balancing step is crucial to prevent well-studied hormones with a large number of gene associations in HGv1 from unduly skewing our model predictions [Fig. 1 (inset histogram)].

Since a unified database of source and target genes for known hormones was not available, we expressly assembled such a database for 51 endocrine hormones, primarily ones listed in a Endocrine Society website (see Section 4), by integrating data from several sources (Dönitz and Wingender, 2014; Rashid *et al.*, 2009; The Gene Ontology Consortium, 2018). We manually went through all Gene Ontology (GO) (The Gene Ontology Consortium, 2018) term names mentioning a given hormone and identified the GO terms that could be unambiguously added to source and target sets for the hormone (Table 1). Every GO term we considered represents a species-agnostic biological process or molecular function, and can hence be tailored to any species by taking the appropriate set of genes annotated to the term. We compiled a human dataset HGv1.human of about 2000 hormone–gene associations (Table 2) derived from the appropriate GO terms and augmented with primary genes (genes encoding a peptide hormone or hormone-binding receptors) from other sources (Dönitz and Wingender, 2014; Rashid *et al.*, 2009). Similarly, we constructed HGv1.mouse dataset by collecting mouse genes annotated to the GO terms collected in the first phase, and by taking the mouse homologs of the primary human genes. This work focuses on HGv1.human (simply referred to as HGv1 in the text), with HGv1.mouse dataset being used to study generalizability of our models.

**Table 1.** Example snapshot of our HGv1 dataset: glucagon and adiponectin hormones along with their dominant source/target tissue(s) and GO terms

Hormone source **→** target tissue(s)	Source terms	Target terms
Glucagon pancreas → liver	GO: 0070091 (glucagon secretion)	GO: 0033762 (response to glucagon)
		GO: 0004967 (glucagon receptor activity)
	GO: 0120116 (glucagon processing)	GO: 0031769 (glucagon receptor binding)
Adiponectin adipose → skeletal muscle, ooctyes, pre-implantation embryos	GO: 0070162 (adiponectin secretion)	GO: 0055100 (adiponectin binding)
		GO: 0033211 (adiponectin-activated signaling pathway)

**Table 2.** HGv1 dataset overview: summary of the 2009 hormone–gene associations in our HGv1.human dataset

Number (#) of hormones/genes	51/1453
Mean # of genes associated with a hormone	39.39 (±78.37)
Mean # of hormones associated with a gene	1.38 (±0.87)
# of source/target genes across all hormones	519/1082
Mean # of source genes for a hormone	12.9 (±32.03)
Mean # of target genes for a hormone	28.24 (±53.66)

*Note*: ± denotes standard deviation here and elsewhere in the text.

### 2.2 Word embeddings are informative of relationship among hormones and genes

**Fig. 2.**
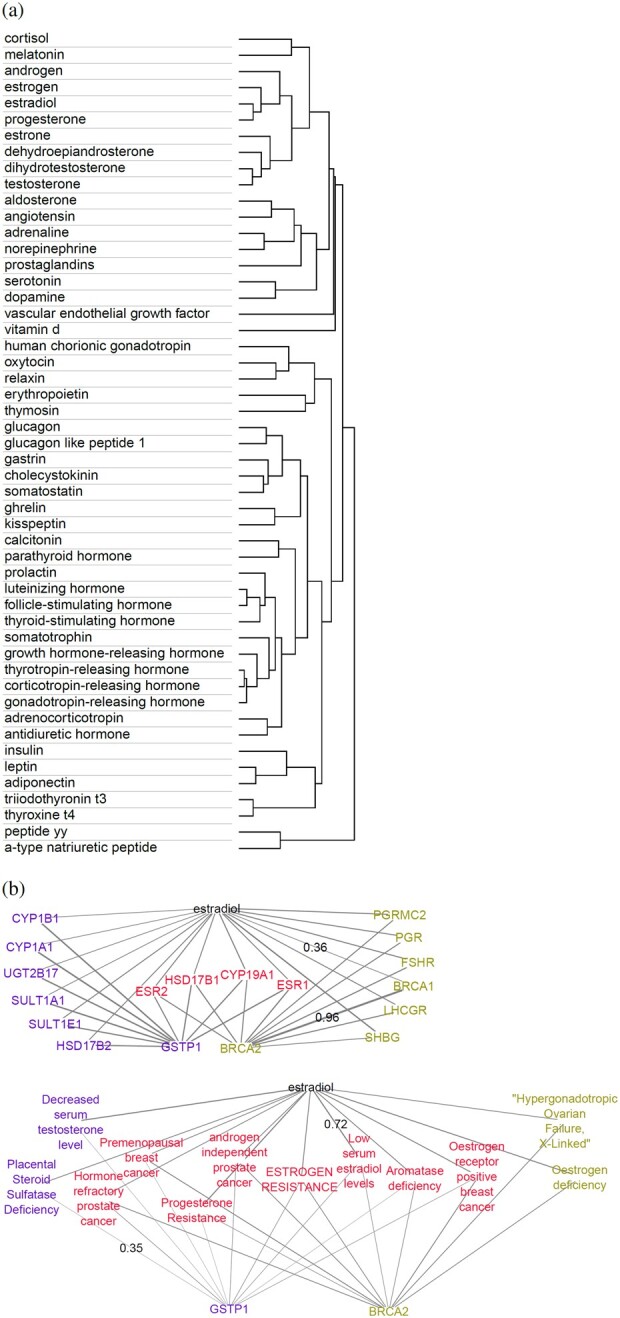
Similarity of hormone embeddings and hormone–gene context: (**a**; top) hierarchical clustering dendrogram of the 200D hormone embeddings using complete linkage method and one minus cosine similarity (cosine of the angle between two vectors) as the distance measure. (**b**; rest) For the predicted hormone–gene pair (estradiol—GSTP1, and estradiol—BRCA2), the gene symbols (middle) or disease terms (bottom) that exhibit cosine similarity of at least 0.35 with the predicted hormone or gene are shown. Cosine similarity is indicated proportionally by edge thickness, with maximum and minimum values shown alongside the corresponding edges

We first assess the quality of literature-based BioWordVec (Zhang *et al.*, 2019) embeddings of hormone names and gene symbols using simple unsupervised learning methods. Hierarchical clustering of the word embeddings of the 51 hormones in our HGv1 dataset (Fig. 2a) revealed that functionally similar hormones often group together into clusters—for instance, neurotransmitter hormones like serotonin and dopamine are clustered together; and so are steroid hormones with sexual and reproductive functions, such as testosterone, estradiol and progesterone.

To evaluate the quality of embeddings of both hormones and genes, we predicted hormone–gene associations using the popular cosine similarity measure between word embeddings of a hormone and a gene, and obtained an average area under the receiver operating characteristics curve (ROC-AUC) of 0.69 (Supplementary Fig. S1). This result confirms the good quality of BioWordVec embeddings, seen in earlier studies on extracting relations among proteins and drugs (Zhang *et al.*, 2019), in our new context of extracting hormone–gene relations from biomedical literature. This result also provides a baseline performance from a simple unsupervised method, against which we can compare the performance of our supervised BioEmbedS model. Furthermore, cosine similarity can aid interpretability of the hormone–gene pairs predicted by BioEmbedS—for instance, BioEmbedS-predicted genes *GSTP1* and *BRCA2* for the hormone estradiol, and Figure 2b shows how similar the embeddings of key estradiol receptor genes (*ESR1* and *ESR2*) and estradiol-related disorders are to both the hormone and the predicted genes’ embeddings.

### 2.3 BioEmbedS strategy on disease–gene predictions is competitive with other methods

Due to lack of existing tools for hormone–gene predictions and due to several tools available for disease–gene predictions, we first validated our BioEmbedS strategy (of an SVM classifier trained on word embeddings) on predicting disease–gene associations from a corpus called EU-ADR (Erik *et al.*, 2012). For performance comparison, we used results reported by the BioBERT, BeFree and Joint Ensemble methods discussed before in Section 1; and our BioEmbedS approach is able to obtain a comparable *F*1-score of 85.84% relative to these methods (Table 3). This is promising as our approach, originally conceived for predicting hormone–gene links, performs reasonably well for a disease–gene prediction task. We do not delve further into these specific disease–gene predictions, as our main focus is to predict hormone–gene relations mediating inter-tissue signaling.

**Table 3.** Performance on disease–gene predictions: our BioEmbedS approach’s 10-fold CV-based result is compared against existing methods’ reported results on the EU-ADR disease–gene corpus

Model	Precision (%)	Recall (%)	*F*1-score (%)
BioEmbedS	77.13 ± 1.41	96.84 ± 2.67	85.84 ± 1.26
BioBERT v1.0	**81.05**	93.90	**86.51**
BioBERT v1.1	77.86	83.55	79.74
BeFree	75.10	97.70	84.6
Joint Ensemble learning	76.43	**98.01**	85.34

*Note*: BioBERT results are obtained from Lee *et al.* (2020); results on BeFree and Joint Ensemble learning models are obtained from Bravo *et al.* (2015) and Bhasuran and Natarajan (2018), respectively.

Bold-faced values indicate the best performance achieved in the corresponding column.

### 2.4 BioEmbedS predicts hormone–gene pairs reliably, and better than available alternatives

In our 5-fold cross-validation (CV) framework described in Methods, an SVM model with a third-degree polynomial kernel was chosen as a consistent classifier, and the resulting BioEmbedS models predicted hormone–gene associations with a reasonably good accuracy of 70.4%±1.8% and *F*1-score of 71.4%±2.7% (Supplementary Table S1). The SVM-based model also achieved better or comparable results than other classifier choices, such as logistic regression and decision trees (Supplementary Table S2). We also note that in our baseline comparison, our supervised BioEmbedS performed better than the unsupervised cosine similarity approach seen above (Supplementary Fig. S1).

**Fig. 3.**
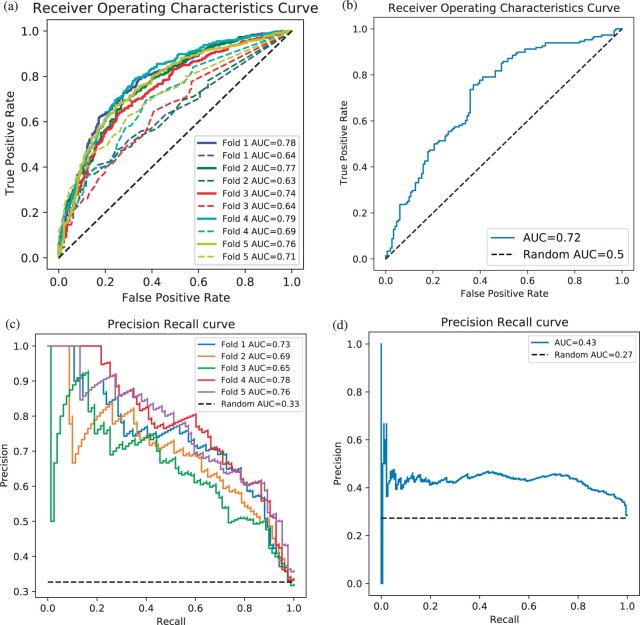
Performance of our BioEmbedS* models in different settings: (**a**) ROC curves of BioEmbedS (solid lines) and STRING (dashed lines) for hormone–gene predictions based on 5-fold CV. (**b**) ROC curve of BioEmbedS for unseen external hormones’ predictions. (**c**) PR curves of BioEmbedS-TS for source/target gene predictions based on 5-fold CV. (**d**) PR curve of BioEmbedS-TS for unseen external hormones’ predictions. AUC of a perfect classifier is one, and of a random classifier is 0.5 for ROC curves. Random classifiers are denoted by black dashed lines in these plots

Since there are no direct hormone–gene prediction tools available to which we can compare our method, the closest alternative was to match a (peptide) hormone to its primary gene encoding the hormone, and then use predicted associations of this gene to other genes. Predicted protein–protein and corresponding gene–gene associations are available in a widely used resource called STRING (Szklarczyk *et al.*, 2015), and we found that our BioEmbedS’ hormone–gene scores were consistently better than STRING’s literature-mining-based scores for the corresponding (mapped) gene–gene pairs (Fig. 3a). We reiterate that STRING is not a direct hormone–gene prediction tool, rather it serves as a generic literature-mining baseline that is used to test if BioEmbedS can predict hormone–gene relations from literature relatively better. To illustrate a bit more with insulin hormone as an example, whereas BioEmbedS would directly predict ‘Insulin–gene’ relations, STRING would predict ‘INS–gene’ associations where INS is the primary gene encoding the peptide hormone insulin, and using gene–gene scores from its ‘text mining’ evidence channel alone (i.e. ignoring STRING’s other evidence channels based on genomic context, experimental assays, etc., to allow STRING to be used as a literature-mining baseline; see Section 4 for more details).

We also evaluated our BioEmbedS model using word embeddings from the state-of-the-art BioBERT model (Lee *et al.*, 2020) as input features. BioEmbedS using our default BioWordVec embeddings performed better than using the original BioBERT embeddings having 768 dimensions (downloaded from https://github.com/naver/biobert-pretrained) and the same embeddings reduced to 200 dimensions to match the dimension of BioWordVec embedding vectors (Supplementary Table S3).

### 2.5 BioEmbedS-TS classifies source versus target genes across different classes of hormones

For genes known to be associated with a hormone, we next classify it they are source or target genes for the hormone. The performance of our BioEmbedS-TS model was also reasonably good with accuracy of 79%±1.9%, and *F*1-score of 84.8 ± 1.3% for target genes and 66 ± 3.5% for source genes (see Table 4 and Fig. 3c). Superior performance in predicting target genes could be because of the disparity between the number of genes in the source and target sets. Table 2 shows that in source versus target classification, source genes constitute the minority class leaving us with fewer samples in the training data to learn underlying patterns.

**Table 4.** BioEmbedS-TS results: classification of source versus target genes across the five test sets

Test fold	Gene type	Precision	Recall	*F*1-score	Accuracy	ROC-AUC	PR-AUC	Kappa score
1	Target	84	88	86	80	0.84	0.73	0.54
	Source	72	64	68	–	–	–	–
2	Target	83	87	85	79	0.83	0.69	0.49
	Source	69	60	64	–	–	–	–
3	Target	82	84	83	76	0.8	0.65	0.44
	Source	63	59	61	–	–	–	–
4	Target	86	85	86	81	0.86	0.78	0.56
	Source	69	71	70	–	–	–	–
5	Target	84	84	84	79	0.86	0.76	0.51
	Source	67	67	67	–	–	–	–

To test if hormones with a large number of associated genes dominated our results, we grouped the hormones into bins based on the number of each hormone’s associated genes and calculated the bin-wise performance for BioEmbedS-TS as well as BioEmbedS. These results, reported in Supplementary Table S4 for BioEmbedS (with hormone-wise details in Supplementary File D1) and Supplementary Table S5 for BioEmbedS-TS, show that bin-wise performance is similar to the overall performance for most bins, thereby suggesting that our BioEmbedS* models perform well across a range of hormones.

### 2.6 BioEmbedS* models generalize reasonably to unseen hormones and well to another species

While previous results are already based on an unseen test set of hormone–gene links performed within a sound 5-fold CV framework, we also wanted to test how well our models would predict for fresh hormones that were never seen in the training/testing part of the CV framework. In other words, we tested BioEmbedS on an independent dataset of hormone–gene pairs pertaining to ‘unseen’ external hormones that were not used to train/evaluate the model (as these hormones had too few gene associations to be considered eligible in the final model building step and hence also every inner/outer CV iteration; see Materials and methods for details). This dataset contained 17 hormones forming 151 associated hormone–gene pairs and 148 non-associated hormone–gene pairs. Although, this dataset does not belong to the same distribution as the one used to train our model due to very few gene associations per hormone, BioEmbedS performed reasonably on this dataset obtaining accuracy of 65%, *F*1-score of 59% and ROC-AUC of 0.72 (Fig. 3b). Similarly, we applied BioEmbedS-TS on a set of hormone–source/target gene pairs from 40 unseen hormones, forming 501 hormone–target gene pairs and 188 hormone–source gene pairs. It was able to correctly classify these pairs with 69% accuracy and area under precision-recall curve (PR-AUC) of 0.43 (Fig. 3d). This PR-AUC of 0.43 is a drop from the average 0.722 seen in CV assessments in Table 4, however, it is higher than the random classifier’s PR-AUC of 0.27 (Fig. 3d), and hence in this regard, our model generalizes reasonably to unseen hormones with few known gene associations.

**Fig. 4.**
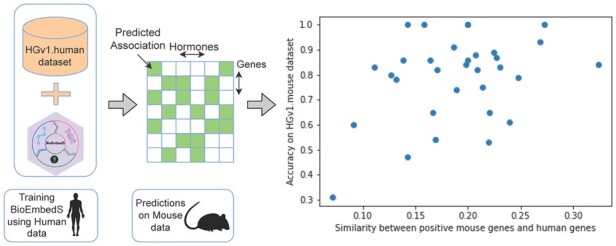
Cross-species translatability of BioEmbedS: accuracy of our HGv1.human-trained BioEmbedS model on each hormone in the HGv1.mouse dataset is plotted against the Jaccard similarity between the known human and mouse gene symbols of the hormone (i.e. the hormone’s positive associated genes in the HGv1.human/mouse datasets, after converting gene symbols to lower-case)

We also assessed how well our model trained on one species (human) generalizes to make predictions in another species (mouse). That is, we applied BioEmbedS model trained on hormone–gene pairs from the HGv1.human dataset (referred to as HGv1 so far) to predict mouse hormone–gene associations. Our BioEmbedS model was able to predict hormone–gene relations in the HGv1.mouse dataset at a reasonable accuracy of 71% and *F*1-score of 73%. Similarly, when BioEmbedS-TS model trained on HGv1.human was used to classify hormone-associated genes in mouse into source and target genes, we achieved 83% accuracy, and a *F*1-score of 78% for source genes and 87% for target genes. Since many human and mouse homologs have identical gene symbols (after converting to lower-case, which is also done prior to getting word embeddings), we wanted to test if the good cross-species performance of our models could be driven solely by gene-symbols-based similarity of the HGv1.human and HGv1.mouse datasets—Figure 4 shows this is not the case. These results together show that our BioEmbedS* models trained using human data generalize well to an organism other than human.

### 2.7 Novel gene predictions are enriched for the corresponding hormone-related diseases, and experimentally validated hormone-responsive genes

**Fig. 5.**
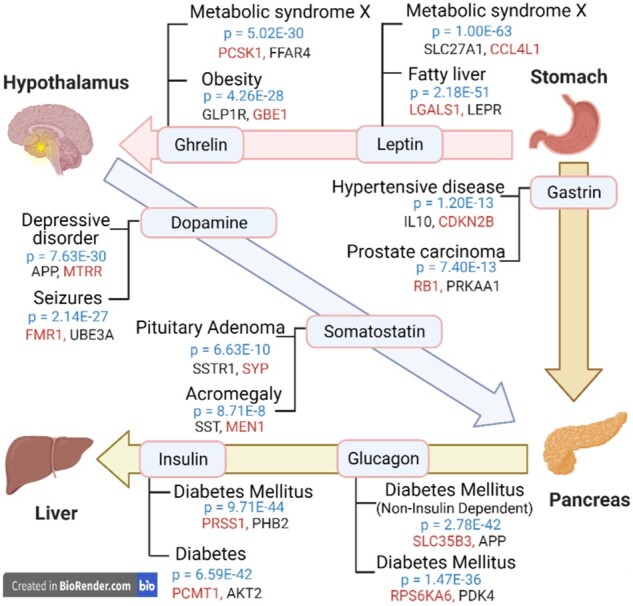
Inter-tissue communication: example of a multi-tissue system with inter-tissue edges indicating hormonal signaling. BioEmbedS predictions for different hormones are enriched for the indicated diseases (top two are shown, along with disease enrichment *P*-values). Shown alongside each tissue–tissue link are examples of known disease genes that are also genes we predicted for a hormone (with darker-shade black marking the genes in HGv1 dataset, and lighter-shade red the novel out-of-HGv1 genes). The hormones shown may have other source→target tissue pairs besides the tissue pair shown here (A color version of this figure appears in the online version of this article.)

The promising performance of BioEmbedS seen so far encouraged us to apply BioEmbedS to predict association between each hormone in HGv1 and all 19 318 human protein-coding gene symbols (Braschi *et al.*, 2019). We were able to predict many novel hormone–gene links not captured in HGv1, comprising new links to any of the 1453 genes in HGv1 (Table 2) or the remaining ‘out-of-HGv1’ genes, which were never seen during training/testing of our model. To validate these predictions, we tested whether the set of predicted genes for a hormone (at a default SVM probability score cutoff; see Section 4) was enriched for diseases already known [according to Piñero *et al.* (2020)] to be related to the hormone. We found this was indeed the case for 16 of the 34 primary hormones (Supplementary File D2a, with primary indicating hormones in HGv1 considered eligible for training the final model as defined in Section 4), and 9 of the 17 unseen external hormones (Supplementary File D2b, with unseen referring to the remaining hormones in HGv1 with very few gene associations). These results pertaining to hormones affecting a subset of tissues is shown in Figure 5. For instance, all the insulin predicted genes are indeed significantly enriched for ‘Diabetes Mellitus’, a disease term that is also recorded as insulin-related in the two hormone–disease ‘ground-truth’ sources that we considered [Endocrine Society (https://www.hormone.org) and DisGeNET (Piñero *et al.*, 2020) resources; see Supplementary File D2a].

**Fig. 6.**
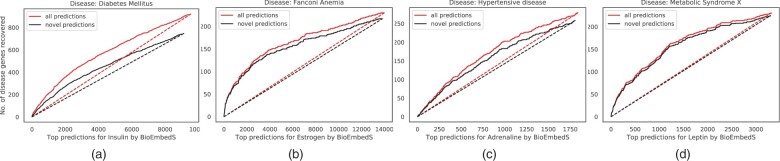
Disease enrichment in novel gene predictions: curves showing the number (no.) of known disease genes (as per DisGeNET; *y*-axis) recovered in top-*k* predicted genes (as per SVM score ranking; *x*-axis) of the corresponding hormone; focusing on all (lighter-shade red) versus novel (darker-shade black) predicted genes of the hormone. Our model (solid curves) performs better than chance recovery of disease genes by a random classifier (dashed lines). Only genes predicted for a hormone with SVM score>0 are considered here; all protein-coding genes are considered in Supplementary Figure S2 (A color version of this figure appears in the online version of this article.)

For well-studied hormones, such as insulin, we repeated the above analysis on only the novel predictions (i.e. predicted hormone–gene links not in HGv1) to test if the disease term enrichments were driven not just by known hormone-specific genes in HGv1 but also by the novel predicted genes. For insulin, BioEmbedS-predicted genes overlapped with 691 of the 1507 Diabetes Mellitus genes recorded in DiSGeNET (disease enrichment $P=9.55\times 10^{-40}$), and 534 of these 691 overlapping disease genes were novel predictions (disease enrichment $P=4.85\times 10^{-9}$). This trend of enrichment of predicted novel genes for the corresponding diseases can also be seen visually in Figure 6 across a range of cutoffs applied on the SVM score to call predictions—specifically, the curve for novel gene predictions is better than that of a random classifier for insulin and other hormones, and follows closely the overall gene-curve for the most part. Furthermore, a more stringent subset of novel BioEmbedS predictions involving totally unseen out-of-HGv1 genes also got validated by a similar disease enrichment analysis (Supplementary Fig. S2).

Besides disease enrichment analysis, we can also test our novel hormone–gene predictions using a more direct evidence: experimentally validated ‘reference’ set of genes linked to a hormone. Experimental studies aimed at finding target rather than source genes of hormones are simpler to conceive, since a single transcriptomic (microarray or RNAseq) experimental study can reveal all the genes that are up/down-regulated in specific cell lines in response to treatment with a hormone. We could find such published experiments and associated hormone-responsive reference genesets for two of the four hormones in Figure 6 (see Section 4). For both these hormones, insulin and estrogen, the novel target gene predictions made by our BioEmbedS* models did indeed overlap with the tested experimentally derived reference genesets with high statistical significance (Table 5).

**Table 5.** Experimental validation of novel predictions: predicted (pred.) gene set refers to the set of novel (out-of-HGv1) target genes of a hormone predicted by our BioEmbedS* models (at default SVM probability cutoffs; see Section 4), and we compute its overlap with an experimentally derived reference (ref.) set of hormone-responsive differentially expressed genes (DEGs, including both up/down-regulated genes) already available for two well-studied hormones via gene expression omnibus (GEO) and Enrichr resources

Hormone	Ref. geneset name (data source and identifiers)	Ref. experiment description (Condition 1 versus 2 in DEG analysis)	Ref. geneset size	Pred. geneset size	Overlap size (overlap *P*-value)
Insulin	Insulin Receptor Associates Promoters GSE107336 (Hancock *et al.*, 2019) (GEO DEG FDR 5% via subseries GSE107334)	HepG2 cells treated with 10 nM insulin versus no insulin for 4 h	4137	3424	1002 ($2.74\times 10^{-33}$)
Insulin	Insulin Receptor Associates Promoters GSE107336 (Hancock *et al.*, 2019) (Enrichr geneset)	HepG2 cells treated with 10 nM insulin versus no insulin for 4 h	457	3424	103 (0.00471)
Estrogen	Estrogen human MCF-7 cells GSE11324 (Carroll *et al.*, 2006) (Enrichr genesets ligand: 89,90,91)	MCF7 cells exposed to 100 nM estrogen for 3, 6 or 12 h versus 0 h	953	9276	607 ($1.40\times 10^{-23}$)
Estrogen	Estradiol human estrogen receptor (ER)-positive MCF7 breast cancer cells GDS3217 (Lin *et al.*, 2007) (Enrichr genesets ligand: 39,40,41)	MCF7 cells exposed to 10 nM estradiol versus vehicle-only at 12, 24 and 48 h	869	9276	487 ($7.54\times 10^{-7}$)

*Note*: GEO study identifiers are prefixed by ‘GSE’ or ‘GDS’. For insulin, besides the ref. geneset from Enrichr, the DEG table in GEO was used to derive another ref. geneset for the same experimental study at 5% false discovery rate (FDR). For estrogen, the DEGs in all timepoints of a study are combined to get a single ref. geneset per study.

## 3 Discussion

This work elucidates the computational problems and challenges in the emerging area of inferring cross-tissue signaling interactions from biomedical literature, and presents a first approach BioEmbedS to specifically predict hormone–gene associations from biomedical literature with reasonably good absolute accuracy, and also comparable or better performance than other popular alternatives, such as STRING. Our BioEmbedS and BioEmbedS-TS models are enabled by a ground-truth dataset HGv1 that we carefully compiled and balanced across different stratifications of the training data in the space of mapped word embeddings of hormone names and gene symbols. The better performance of our method over other alternatives follows from our models being the first systematically developed ones to make hormone–gene predictions, and other methods being literature-mining-based relation extraction methods that are generic or designed for other prediction tasks, such as disease–gene prediction.

Our HGv1 dataset can be viewed as a two-layered/bipartite graph (with HGv1 hormone–gene relations being the edges/links between nodes in the hormone layer and the gene layer), hence our hormone–gene prediction task can be viewed as a link prediction problem in bipartite graphs (Kunegis *et al.*, 2010). Current link prediction methods for bipartite or more general graphs (Kumar *et al.*, 2020; Lü and Zhou, 2011), such as ones based on pairwise node similarity, utilize attribute data available at the nodes and/or the structural connectivity/neighborhood of nodes based on existing links in the graph to predict new links. We decided to utilize only the attribute data (word embeddings) of nodes to do hormone–gene bipartite link prediction, since our HGv1 graph does not have rich structural connectivity. In detail, a large number of genes in our HGv1 dataset (1102 of the 1453) are uni-hormone genes connected to only one hormone, and removing the few other multi-hormone genes disconnects the HGv1 bipartite graph into several connected components (one per hormone).

A caveat in this work worth mentioning is that randomly selected genes for a hormone need not be truly negative examples. Our model may also predict false associations based on high co-occurrence or context word similarity but no functional relationship, and a careful set of negative examples can help mitigate this issue. Our balancing strategy using Synthetic Minority Oversampling Technique (SMOTE) is also not without its pitfalls, especially when applied to high-dimensional data (Fernández *et al.*, 2018), and we coupled it with under-sampling to carefully balance our training data across different hormones that are represented in the literature to different extents and to address imbalance in the number of source versus target genes. Also, we use relatively well-studied ‘primary’ hormones (with a higher number of known hormone-gene associations) for training the model. So when validating the method on independent test set comprising poorly studied hormones with fewer known hormone–gene associations, there is an expected drop in performance. Nevertheless, reasonably good accuracy of our model on different unseen test sets and on an organism other than human, and our disease enrichment and experimental validation analysis of novel gene predictions taken together suggest that our model predictions are indeed generalizable to make novel predictions about inter-tissue gene signaling mediated by a hormone.

Future work could focus on integrating literature information with independent omics data, such as multi-tissue genomic data (The GTEx Consortium, 2020) or rapidly accumulating single-cell/spatial transcriptomics data (Armingol *et al.*, 2021). This literature+omics integration could not only improve model performance, but also help interpret further our novel hormone–gene predictions. For instance, a location (tissue or celltype) where a hormone–gene association happens could be predicted using the corresponding location’s omic data. As another example, systematic interpretation/validation of non-coding gene predictions for hormones [such as the preliminary long non-coding RNA (lncRNA) predictions, we provide in our website] using independent omics data could potentially reveal unexplored routes of inter-tissue communication. Finally, a special feature of our ground-truth hormone–gene dataset HGv1 is its applicability to organisms beyond human and mouse as it is based on species-agnostic GO terms, and this bodes well for extending our work to other multi-cellular organisms in the future. We hope our work on inter-organ hormone–gene network models, demonstrated concretely for the human and mouse organisms, stimulates more work along these lines on cross-tissue signaling for different organisms and thereby advances the field of whole-body, cross-tissue gene network modeling.

## 4 Materials and methods

### 4.1 Our BioEmbedS and BioEmbedS-TS approach

**Word-embeddings-based classifiers**: To proceed with the classification models, we need to represent our data (genes and hormones) in a way that can be used by a classifier. There are several ways of doing it, the most recent and efficient methods being from the deep learning community, known as word embeddings. Specifically, we use word embeddings for gene symbols and hormones from a FastText model pre-trained on the PubMed biomedical corpus called BioWordVec (Zhang *et al.*, 2019) (BioWordVec model/embeddings are downloaded from https://github.com/ncbi-nlp/BioSentVec). FastText (Bojanowski *et al.*, 2017) is a neural network model learned by minimizing cross-entropy loss between each word and its predicted context within a fixed window size. We used 200D embeddings obtained using a skip-gram-based implementation with window size 20. This ensures the embedding vectors of not only co-occurring entities in a document but also entities with similar word neighborhoods exhibit high similarity (Park *et al.*, 2019). BioWordVec actually uses subword information to obtain embeddings of words (including out-of-vocabulary words), i.e. the embedding of each word is represented by the sum of embeddings of all *n*-grams (3 ≤ *n* ≤ 6) in the word (after converting the words, including hormones and gene symbols, to lower-case) (Bojanowski *et al.*, 2017; Zhang *et al.*, 2019). Please note that BioWordVec is already trained using the PubMed corpus and so our HGv1 dataset is not used to obtain the word embeddings; HGv1 (training/testing splits) is instead used along with the pre-trained word embeddings to build our BioEmbedS* classifiers.

Existing studies on prediction of disease–gene associations have shown that SVM classifiers perform well when used with word embeddings (Bhasuran and Natarajan, 2018). So we explored SVM along with Random Forest (RF) classifiers as our primary classifiers, and compared them with other secondary choices of classifiers as well, and found SVMs to perform well (see Section 2). Hence, we decided on an SVM-based model to predict hormone–gene relations and call it BioEmbedS (with S denoting SVM). Similarly, we use an SVM-based model to classify source versus target genes for associated genes for a given hormone and call it BioEmbedS-TS (with -TS denoting Target versus Source).

**Stratified/nested CV**: We explored the parameter space (both hyper-parameter tuning and parameter learning) of our SVM and RF models using a 5-fold CV strategy that is stratified to ensure even distribution of each hormone’s genes across the different folds, and nested to allow proper partitioning of the HGv1 data into training, validation and test sets. In detail, a stratified split amounts to considering each hormone with a certain number of associated genes in HGv1 (denoted *n*), and putting randomly chosen $\left\lfloor \frac{n}{5}\right\rfloor$ genes into each of the 5-folds, and the remainder genes randomly (one each) into any of the 5-folds. This procedure done for the BioEmbedS model ensures that each fold has genes belonging to each hormone proportional to their presence in the overall dataset. A similar procedure to distribute the number of source (and separately target) genes of each hormone evenly across the 5-folds was done for the BioEmbedS-TS model also. For BioEmbedS, we also created a negative class (non-associations) dataset of the same size across hormones and folds as the positive class (hormone–gene associations) dataset described above. In detail, for each hormone, we excluded its set of positive/associated genes (of size denoted by *n*) from all genes present in HGv1, and randomly chose from the remaining genes a set of *n* ‘negative’ genes, which are then split across the five CV folds in the same manner as the positive genes for this hormone. In the nested 5-fold CV strategy that we use to build both BioEmbedS and BioEmbedS-TS models, we make a train/validation/test split using 3/1/1-folds, respectively, and identify the classifier’s parameters by using only train and validation splits. The test split is kept aside from the training process, and used solely to report the model performance.

**Over/under-sampling (balancing) training set embeddings**: Our HGv1 dataset has a skewed distribution of the number of genes associated with different hormones [Fig. 1 (inset histogram); Table 2]; so it is important to prevent well-studied hormones with a large number of gene associations in HGv1 from unduly influencing our model. To address the problem of class imbalance, oversampling techniques like SMOTE (Chawla *et al.*, 2002) synthesize new examples from existing ones for the minority class, whereas under-sampling techniques like Condensed Nearest Neighbours (Hart, 1968), TOMEK Links (Tomek, 1976), etc. selectively remove examples from the majority class. A combination of oversampling and under-sampling techniques is shown to perform better than using either alone (Chawla *et al.*, 2002). We strategically apply a combination of SMOTE and TOMEK Links on the mapped embeddings of only genes with exactly one hormone association in HGv1. Working in the space of embeddings of such ‘uni-hormone’ genes is both desirable in facilitating SMOTE oversampling of our data, and permissible as a large fraction of all genes in HGv1, 1102 of all 1453, are uni-hormone (i.e. associated uniquely with some hormone, as opposed to being multi-hormone or associated with more than one hormone within HGv1). This technique applied to BioEmbedS and a similar technique applied to BioEmbedS-TS handle the large variation in HGv1 in the number and type (source versus target) of genes respectively across hormones.

For BioEmbedS, we assign a unique class ID to every hormone, and all the genes uniquely associated with the hormone belong to the class indicated by its class ID (with all multi-hormone genes discarded). We then use a combination of SMOTE oversampling and TOMEK Links under-sampling [with the number *k* of nearest neighbors in SMOTE set to two in the implementation used (Lemaître *et al.*, 2017)] to get an approximately equal number of genes/examples (same as the highest number of genes associated with a hormone among all the hormones in HGv1) for every hormone. We now have a dataset with approximately equal numbers of the genes related to every hormone that forms hormone–gene pairs of positive class for our binary classification problem. The following strategy is applied to create the negative class (set of non-associated hormone–gene pairs). For every hormone, we construct a set that contains the ‘genes’ (synthesized examples from oversampling and under-sampling) associated with all the hormones except the one under consideration. We randomly select from this negative set as many examples as in the positive set of this hormone, in order to maintain class balance between the positive and negative associations for the hormone. Repeating this process for each hormone finally results in a dataset that is balanced across the different hormones and positive/negative class.

For BioEmbedS-TS, we define two unique IDs for every hormone; one maps to the source genes and the other maps to the target genes for that hormone (again all multi-hormone genes, defined exactly the same way as above for BioEmbedS, are discarded, along with the small set of genes that are annotated as both source and target for the same hormone). The source and target genes associated with a hormone belong to classes indicated by their IDs. This makes the number of classes equal to twice the number of hormones present in our dataset. We use SMOTE and TOMEK Links strategy as for BioEmbedS to get an approximately equal number of source and target genes (same as the highest number of source/target genes associated to a hormone among all the hormones in HGv1) for every hormone. Finally, we have a dataset that is balanced across the different hormones and source/target class.

**Putting it all together—balancing and model selection within nested CV**: The overall careful application of our balancing and model selection steps to the 3/1/1 train/validation/test folds is shown in Algorithm 1, along with what constitutes the training and testing sets of each inner/outer CV iteration (e.g. validation and test folds are the respective testing sets for inner and outer CV loops). As for the genes considered by Algorithm 1, training sets are restricted to contain only uni-hormone genes to facilitate application of SMOTE and TOMEK Links as discussed before, whereas testing sets are allowed to contain both uni- and multi- hormone genes to reflect a real-world setting where any gene may be queried for its association to a hormone. As for the hormones considered for the BioEmbedS model, the overall algorithm considers only hormones with at least five gene associations to permit 5-fold CV; additionally, each inner/outer CV iteration considers only hormones with sufficient gene associations as ‘eligible’ for further analysis. In detail, every hormone with at least three associations to uni-hormone genes in an iteration’s training set is considered eligible for this iteration (to permit two-nearest-neighbor SMOTE), and the remaining hormones are removed from this iteration’s training as well as testing sets. Similarly for the BioEmbedS-TS model, hormones with at least five source and five target gene associations are considered for 5-fold CV. In each inner/outer CV iteration’s training set, only the hormones with at least three uni-hormone–source gene and three uni-hormone–target gene associations are considered ‘eligible’, and the remaining hormones are removed from training as well as the test set for this iteration.

SVM and RF models with different hyper-parameter combinations are trained on the resulting balanced training folds, and Cohen’s Kappa score of these trained models on the validation folds is used to select the best model. In each iteration, the test fold is never used for training or choosing hyper-parameters for the classifier. For BioEmbedS, an SVM model with a third-degree polynomial kernel was chosen as the best classifier by Algorithm 1 (Step 10) consistently in all five outer CV iterations (Supplementary Table S1). This SVM model was later trained using the entire HGv1 dataset, after applying similar restrictions and balancing as in Step 11 of Algorithm 1, to obtain the final BioEmbedS model—this final model was used for making all protein-coding gene predictions (including novel ones discussed in Section 2). Hormones found eligible for building the final BioEmbedS model amounted to 34 and are known as primary hormones hereafter; the remaining 17 ineligible hormones would also have been ineligible (and hence discarded or unseen when choosing/training models) in each of the inner/outer CV iterations of Algorithm 1 due to these hormones’ insufficient gene associations. For BioEmbedS-TS, SVM classifiers with different kernels/hyper-parameters were chosen as the best classifier in the different outer CV iterations of Algorithm 1 (Step 10). Among these best classifiers, we chose the simplest model: an SVM classifier with a third-degree polynomial kernel. We then trained this SVM model using the entire HGv1 dataset of source versus target hormone–gene relations, after applying similar restrictions and balancing as in Step 11 of Algorithm 1, to obtain the final BioEmbedS-TS model—this final model was applied to classify all hormone–gene associations predicted by BioEmbedS as source versus target type (using cutoffs shown below at the end of Section 4). Only 14 hormones with sufficient source and target gene associations were found to be eligible for building the final BioEmbedS-TS model.

Algorithm 1: Pseudocode for nested 5-fold CV.

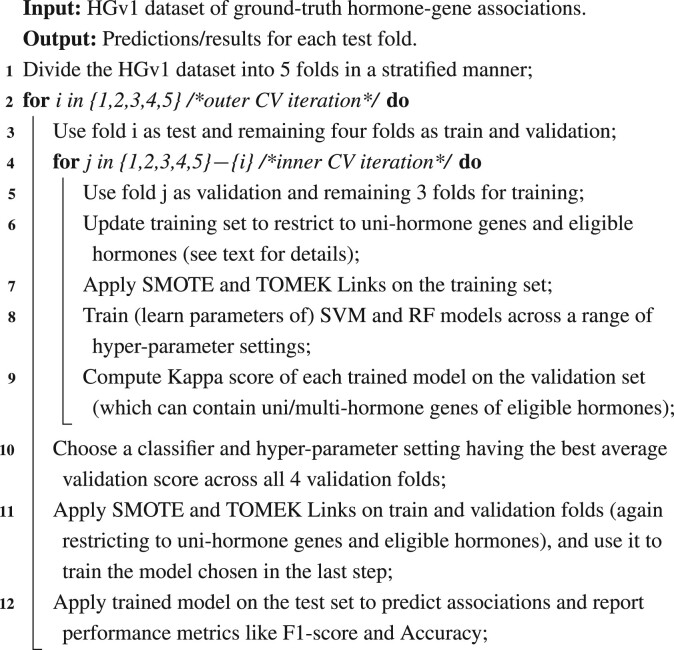

### 4.2 Evaluation framework: performance metrics and enrichment/clustering analysis

We evaluate our model on five different unseen test sets (as shown in Algorithm 1), by reporting the performance of classifiers on these test sets using standard performance metrics like precision, recall, *F*1-score, accuracy, Kappa score, ROC-AUC and PR-AUC. All these metrics take values in the range 0–1 and hence can be expressed as percentages (with the exception of Kappa score that can take negative values), with higher values indicating better performance. Supplementary Methods S1.1 provides definitions for these metrics assuming the two classes in our binary classification problems are as follows: for BioEmbedS, we naturally let positive and negative class to refer respectively to association and non-association of a hormone–gene pair; for BioEmbedS-TS that starts with an associated hormone–gene pair, we arbitrarily let positive and negative class refer to hormone–source gene pair and hormone–target gene pair association, respectively. Given these class definitions, a false positive for instance, for BioEmbedS would be a non-associated hormone–gene pair that is wrongly predicted as associated by our method; and for BioEmbedS-TS would be a hormone–target gene pair that is wrongly predicted as hormone–source gene pair by our method.

Hormone–gene predictions are called using the final BioEmbedS model described above at an SVM probability score of at least 0.7, with this default cutoff value chosen so as to get a reasonable number of predictions for each hormone. To validate the predicted genes of a hormone, we perform a disease enrichment analysis using the Enrichr tool (Chen *et al.*, 2013) and DisGeNET (Piñero *et al.*, 2020) collection of known disease-related genes. All reported disease enrichment *P*-values from this analysis are corrected for multiple testing of different DisGeNET disease terms. For validation of novel predictions, besides enrichment for disease genesets, we also tested for overlap with experimentally derived reference genesets. We obtained these reference sets by searching the names of each of the four hormones in Figure 6 against all terms in the following Enrichr libraries of reference genesets (Chen *et al.*, 2013): ‘Ligand Perturbations from GEO’ (‘down’ and ‘up’ genesets combined, since, we do not make distinction between down/up-regulated target genes in our predictions), and ‘RNAseq Automatic GEO Signatures Human’ (again ‘down’ and ‘up’ genesets combined) (https://maayanlab.cloud/Enrichr/#libraries, accessed Jun 30, 2022). We excluded studies where the hormone was used in conjunction with some other stimuli to treat the cells, and we also chose cell line over disease studies (e.g. for insulin, we preferred HepG2 cell line hormone-treatment over insulinoma-tumor-related studies). These choices helped avoid any unwanted influence from non-hormonal stimuli or disease factors when identifying a hormone’s target genes. Hypergeometic distribution is used to obtain the Overlap *P*-values reported in Table 5.

Also, as part of validating our predictions, we inspected the pairwise overlap between the highly significant gene sets predicted for hormones, and found that biologically related hormones, such as insulin, leptin and glucagon, indeed group together in a hierarchical clustering-based dendrogram (Supplementary Fig. S3), due to their higher pairwise similarities than most other hormone pairs.

In comparison of STRING (Szklarczyk *et al.*, 2015) to our BioEmbedS model within the 5-fold CV framework, STRING is used as a generic literature-mining baseline as it is not a direct hormone–gene prediction tool. We evaluated STRING on the following 14 peptide hormones alone (primary gene encoding the hormone shown in parenthesis): calcitonin (*CALCA*), cholecystokinin (*CCK*), glucagon (*GCG*), insulin (*INS*), leptin (*LEP*), parathyroid hormone/parathyrin (*PTH*), thyrotropin-releasing hormone (*TRH*), antidiuretic hormone/vasopressin (*AVP*), prolactin (*PRL*), somatostatin (*SST*), gastrin (*GAST*), ghrelin (*GHRL*), growth hormone-releasing hormone (*GHRH*) and oxytocin (*OXT*). A ranked list of STRING’s hormone–gene predictions were obtained using the text-mining scores (in the 9606.protein.links.detailed.v11.0.txt file downloaded from the STRING website) of genes associated by STRING to each hormone’s primary gene (and assuming the lowest score of zero for non-associated genes). The same five CV folds derived from the HGv1 dataset were used to evaluate both BioEmbedS and STRING, after excluding positive/negative examples involving (i) hormones that were not eligible in the fold (see Algorithm 1) when evaluating both BioEmbedS and STRING, and (ii) hormones not present in the above peptide hormones list when evaluating STRING alone.

### 4.3 Implementation details and data/code availability

Implementations are done using Python *Scikit-learn* framework using *decision_function()* and *predict_proba()* methods of support vector classification in *sklearn* respectively to obtain the SVM model scores for all hormone–gene pairs and the probability of association between hormone–gene pairs (probability score of SVM). For reproducibility purposes, we provide hyper-parameter choices in Supplementary Methods S1.2 and open-source code and relevant data in a public repository. Specifically, the proposed HGv1 dataset along with our models’ predictions, and the code to reproduce this work are available respectively at the web portal https://cross-tissue-signaling.herokuapp.com/, and code repository https://github.com/BIRDSgroup/BioEmbedS. In the web portal, we show BioEmbedS-predicted hormone–gene associations at the default cutoff (of at least 0.7 for BioEmbedS SVM probability), as well as BioEmbedS-TS-predicted type of the association (i.e. source versus target type for hormones that were used to train the BioEmbedS-TS model; as explained above, hormones with insufficient number of source or target genes were not used to train the BioEmbedS-TS model). In detail, a hormone-associated gene whose BioEmbedS-TS SVM probability is: (i) at least 0.7 is predicted as a source gene for the hormone; (ii) at most 0.3 is predicted as a target gene for the hormone; and (iii) the rest are called as inconclusive or ‘don’t know’ genes. This association type of source or target can then be used to select either the known source or target tissues respectively of the hormone, as recorded in HGv1, to infer the potential locations (tissues) of a hormone–gene association. Our web portal also lists these predicted tissue locations. Furthermore, the target genes obtained using the above default cutoffs were used toward the experimental validation analysis reported in Table 5.

## Supplementary Material

btac578_Supplementary_Data
