# Supplementary material for: Predicting cross-tissue hormone–gene relations using balanced word embeddings
Source: Bioinformatics. 2022 Aug 24;38(20):4771–81. doi: 10.1093/bioinformatics/btac578 (PMC9563690; doi:10.1093/bioinformatics/btac578)
Supplement: btac578_Supplementary_Data [file btac578_supplementary_data.pdf]

Supplementary Information

for the manuscript "Predicting cross-tissue hormone-gene relations using balanced word embeddings"

1 Supplementary Methods 1
 1.1 Performance Metrics 1
 1.2 Hyperparameters of BioEmbedS classifiers 2
 1.3 Assembling the ground-truth dataset HGv1: Web resources 3
2 Supplementary Tables 4
3 Supplementary Figures 7
4 Supplementary Files 10

1 Supplementary Methods

1.1 Performance Metrics

As explained in the main text, the positive and negative class in our binary classification problems refer respectively to: association and non-association of a hormone-gene pair in the BioEmbedS setting, and hormone-source gene pair and hormone-target gene pair association in the BioEmbedS-TS setting. Applying standard definitions to these settings yields the following definitions, with number abbreviated as "#".

True Positives (TP): # of positive hormone-gene pairs predicted as positive by BioEmbedS;
# of hormone-source gene pairs predicted correctly by BioEmbedS-TS.

True Negatives (TN): # of negative hormone-gene pairs predicted as negative by BioEmbedS;
# of hormone-target gene pairs predicted correctly by BioEmbedS-TS.

False Positives (FP): # of negative hormone-gene pairs predicted as positive by BioEmbedS;
# of hormone-target gene pairs predicted as hormone-source gene pairs by BioEmbedS-TS.

False Negatives (FN): # of positive hormone-gene pairs predicted as negative by BioEmbedS;
# of hormone-source gene pairs predicted as hormone-target gene pairs by BioEmbedS-TS.

We evaluate our classifiers on the following performance metrics derived from the above counts.

1. <u>Precision</u>: In the context of BioEmbedS classifier, it represents the proportion of predicted hormone-gene pairs (TP + FP) that are actually correct as per the HGv1 dataset (TP). In the context of BioEmbedS-TS, it indicates the proportion of predicted source genes that are truly the source genes as per the HGv1 dataset.

$$Precision = \frac{TP}{TP + FP}$$

2. <u>Recall</u>: In the context of BioEmbedS, it is the ratio of hormone-gene associations that our model can predict (TP) to the total associations present in the HGv1 dataset (TP

+ FN). In the context of BioEmbedS-TS, it is the ratio of source genes that our model recovers to all source genes present in the HGv1 dataset.

$$Recall = \frac{TP}{TP + FN}$$

3. F1-score: It is the harmonic mean of Precision and Recall scores.
4. Accuracy: It indicates out of all the model's predictions, how many are correct predictions.

$$Accuracy = \frac{TP + TN}{TP + TN + FP + FN}$$

5. Cohen's Kappa score: It indicates how often the model's predictions and the actual HGv1 labels for all considered hormone-gene pairs agree relative to random chance agreement, and is a useful metric for classification with imbalanced datasets [2].
6. ROC-AUC: The area under the *Receiver Operating Characteristics* (ROC) curve, which plots TPR (true positive rate or recall $\frac{TP}{TP+FN}$) on the y-axis against FPR (false positive rate or $\frac{FP}{TN+FP}$) on the x-axis, with different points on the curve based on different cutoffs applied on the model scores to make positive vs. negative class predictions [1].
7. PR-AUC: The area under the *Precision-Recall* (PR) curve, which plots precision on the y-axis against recall on the x-axis, with different points on the curve again based on different cutoffs applied on the model scores to make positive vs. negative class predictions [1].

1.2 Hyperparameters of BioEmbedS classifiers

Besides choosing SVM (Support Vector Machines) and RF (Random Forests) as our primary classifiers for use with the BioEmbedS model (see main text and Suppl Table S1), we also tried other secondary choices of classifiers (see Suppl Table S2). Hyperparameters of these primary and secondary classifiers are given below, and are implemented using the *Scikit-learn* machine learning framework in Python [3]. There were no hyperparameters to choose for simpler models like logistic regression.

SVM: The range of hyperparameter values considered for the SVM classifier are as follows. For kernel functions, we tried RBF (Radial Basis function) and polynomial kernel types. The model complexity parameter C had 9 equally spaced values between -4 to 4 in the log space. Gamma parameter for RBF kernel had 12 equally spaced values between -9 to 2 in the log space. Degree parameter for the polynomial kernel had values 2, 3, 5 and 7. In each fold, polynomial kernel with degree = 3 and $C = 1$, was chosen as the best classifier based on scores on the validation set. We also choose this hyperparameter setting of SVM as our final classifier model to make novel predictions.

RF: The range of hyperparameter settings considered for the sklearn implementation of the RF classifier are as follows. For the number of trees in the forest, we tried 7 arbitrarily pre-selected values from 100 to 1600; and for the maximum depth of each tree, we tried 9 pre-selected values from 120 to 360. We let the minimum number of samples required to be at a leaf node to be 1, 2, or 4; and the minimum number of samples required to split an internal node to be 2, 3, 5, or 7.

Neural Networks: The Neural Networks have 2 hidden layers. The number of units in each layer was sampled among 32, 64 and 128 units. Four values of learning rate were tried out between 0.0001 and 0.1, and the regularisation parameter was chosen among $10^{-3}, 10^{-4}$, and 10^{-5}. From all these possible configurations, the combination of parameters that gave the best results were chosen.

XGBoost: For the XGBoost model, 5 values of learning rate were sampled between 0.03 and 0.3 in the log-space, 5 values of maximum depth were sampled between 2 and 6, and 5 values of the number of estimators were sampled between 100 and 150 in the linear space. From these, the combination of parameters that gave the best results were chosen.

Decision Trees: Five values of maximum depth were sampled between 2 and 6, and five values of the number of estimators were sampled between 100 and 150 in the linear space.

1.3 Assembling the ground-truth dataset HGv1: Web resources

We have already provided a description/overview of how we assembled our HGv1 dataset in Results in the main text. Here we provide additional details about the websites from which certain pieces of relevant information were downloaded and collated. HGv1 contains information about hormones primarily listed in a Endocrine Society website (https://www.hormone.org/your-health-and-hormones/glands-and-hormones-a-to-z, accessed Jul 23, 2019). For information about gene symbols annotated to hormone-related Gene Ontology (GO) terms, HGv1 uses the web resource AmiGO ((http://amigo.geneontology.org/), accessed August 2020). For the HGv1.mouse dataset, the human-to-mouse homology mapping was done via the MGI Batch Query (http://www.informatics.jax.org/batch, accessed Nov 11, 2020). This query yielded one-to-one homology mapping from human to mouse gene symbols for 37 of the 43 source genes and 94 of the 97 target genes.

References

1. Jesse Davis and Mark Goadrich. The relationship between Precision-Recall and ROC curves. In *Proceedings of the 23rd International Conference on Machine Learning*, ICML '06, page 233–240, New York, NY, USA, 2006. Association for Computing Machinery.
2. C. Ferri, J. HernÃ¡ndez-Orallo, and R. Modroiu. An experimental comparison of performance measures for classification. *Pattern Recognition Letters*, 30(1):27 – 38, 2009.
3. F. Pedregosa, G. Varoquaux, A. Gramfort, V. Michel, B. Thirion, O. Grisel, M. Blondel, P. Prettenhofer, R. Weiss, V. Dubourg, J. Vanderplas, A. Passos, D. Cournapeau, M. Brucher, M. Perrot, and E. Duchesnay. Scikit-learn: Machine learning in Python. *Journal of Machine Learning Research*, 12:2825–2830, 2011.

2 Supplementary Tables

Fold	Precision	Recall	F1-score	Accuracy	ROC-AUC	PR-AUC	Kappa Score
1	0.70	0.76	0.73	0.71	0.78	0.76	0.43
2	0.70	0.71	0.70	0.70	0.77	0.76	0.40
3	0.67	0.70	0.69	0.68	0.74	0.71	0.36
4	0.71	0.77	0.74	0.73	0.79	0.76	0.46
5	0.70	0.72	0.71	0.70	0.76	0.74	0.40

Table S1: **BioEmbedS performance across 5 test folds:** Results are using the 5 test folds of our cross validation (CV) framework using the best primary classifier (which turned out to be a SVM classifier with degree-3 polynomial kernel and $C = 1$ as mentioned in Suppl Methods 1.2).

Model	Precision	Recall	F1-score	Accuracy	ROC-AUC	PR-AUC	Kappa Score
Primary classifier (SVM)	0.69	0.73	0.71	0.70	0.77	0.75	0.41
Neural Network	0.68	0.77	0.72	0.70	0.76	0.73	0.41
XGBoost	0.60	0.84	0.70	0.64	0.70	0.66	0.29
Decision Trees	0.66	0.78	0.71	0.68	0.74	0.71	0.37
Logistic Regression	0.52	0.62	0.58	0.53	0.53	0.53	0.05

Table S2: **BioEmbedS performance for different choices of classifiers:** Performance reported is average across the 5 CV test folds – for instance SVM's performance is average of performance reported in Table S1. It is evident that the SVM classifier achieved better or comparable performance relative to other classifiers. It is also clear that simpler models like Logistic Regression could not capture patterns in the dataset, and higher order function approximators like Neural Networks or algorithms like SVM provide better results.

Fold	Precision	Recall	F1-score	Accuracy	ROC-AUC	PR-AUC	Kappa Score
BioBERT (768D)	0.69	0.62	0.65	0.67	0.73	0.72	0.34
BioBERT (200D)	0.67	0.66	0.66	0.67	0.71	0.7	0.33

Table S3: **BioEmbedS performance using different (BioBERT) embeddings:** Original BioBERT word embeddings, each of which is a 768-dimensional (768D) vector, and the same embeddings reduced to 200 dimensions (200D) using Principal Component Analysis (PCA), were used as input features to BioEmbedS instead of our default input features comprising 200D FastText-based BioWordVec embeddings (used in Tables S1,S2). Performance reported is average across the 5 CV test folds.
To ensure fair comparison, the same procedure used for learning the default BioWordVec-BioEmbedS model was followed here to learn the BioBERT-BioEmbedS model – specifically, in each fold, the best classifier out of SVM and Random Forest classifiers were chosen and the hyperparameters for these classifiers were selected by grid search over the same set of values as used for the BioWordVec-BioEmbedS model.

Bin	Bin criteria (#genes linked to a hormone)	#hormones/-#hormone-gene pairs	Precision	Recall	F1-score	Accuracy
1	≤ 5	17.6/90	0.80	0.63	0.70	0.74
2	$> 5, \leq 11$	7/113.2	0.71	0.65	0.68	0.66
3	$> 11, < 99$	7.2/337.2	0.68	0.76	0.72	0.71
4	≥ 99	1/198.4	0.68	0.78	0.72	0.71

Table S4: **Bin-wise results for BioEmbedS:** We divided the hormones into 4 bins taking into consideration the number of genes associated with hormones and report the bin-wise performance of BioEmbedS (specifically average performance across the 5 CV test folds). # denotes "number of" in this and other tables. The numbers reported here are average across the 5 test folds.

Bin	Bin criteria (#genes for a hormone) / #hormones	Gene Type	#hormone-gene pairs	Precision	Recall	F1-score	Accuracy
1	≤ 16/ 7.2	Target	53.4	0.86	0.89	0.88	0.83
		Source	26	0.76	0.69	0.72	
2	$> 16, < 93$/ 2.6	Target	66.4	0.93	0.89	0.91	0.86
		Source	18.2	0.67	0.76	0.71	
3	≥ 93/ 1	Target	55.2	0.72	0.77	0.74	0.68
		Source	37.8	0.62	0.55	0.58	

Table S5: **Bin-wise results for BioEmbedS-TS:** We divided the hormones into 3 bins taking into consideration the number of source and target genes associated with hormones and report the bin-wise performance of BioEmbedS-TS (specifically average performance across the 5 CV test folds). The number of hormones and hormone-gene pairs are also average across the 5 test folds.

3 Supplementary Figures

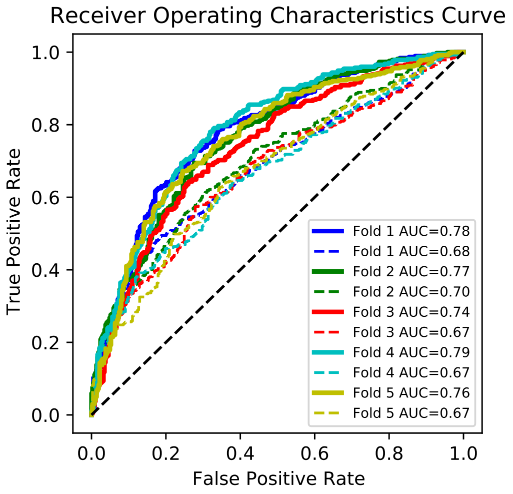

Fig. S1. Cosine similarity and BioEmbedS performance: ROC curves for hormone-gene predictions using unsupervised cosine similarity based method (dashed lines), and our supervised method BioEmbedS based on the SVM classifier (solid lines).

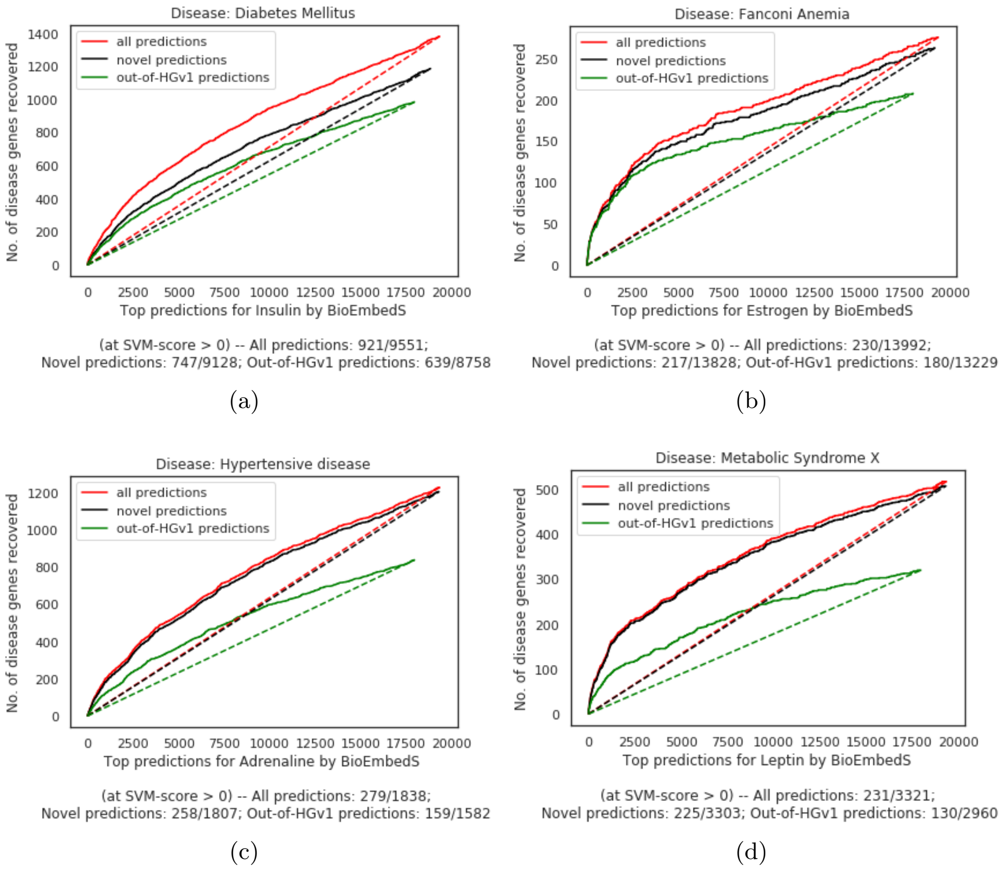

Fig. S2. Disease enrichment in novel and out-of-HGv1 gene predictions: Curves showing the number (no.) of known disease genes (y-axis) recovered in top-k predicted genes (as per SVM score ranking; x-axis) of the corresponding hormone; focusing on all (red) vs. novel (black) vs. out-of-HGv1 (green) predicted genes of the hormone. To clarify these terms for a given hormone, ‘all” refers to predictions made for all 19,318 protein-coding genes; “novel” refers to a subset of all predicted genes such that the hormone-gene **pair** is not in HGv1; and “out-of-HGv1” refers to a subset of the novel predicted genes such that the **gene** is also not in HGv1 (i.e., the predicted gene is not associated with any hormone in HGv1, and hence totally absent from HGv1 and unseen during model building).
Our model (solid curves) performs better than chance recovery of disease genes by a random classifier (dashed lines). Below each hormone’s disease enrichment plot, the number of disease genes overlapping the predicted genes at SVM score > 0 for the hormone is shown as a ratio (# overlapping disease genes / # predicted genes). It is evident that even after removing the hormone-associated HGv1 genes, a significant number of disease-related genes are left and they get predicted towards the top by BioEmbedS (black curves). Moreover, BioEmbedS performs well on the totally unseen out-of-HGv1 genes too (green curves).

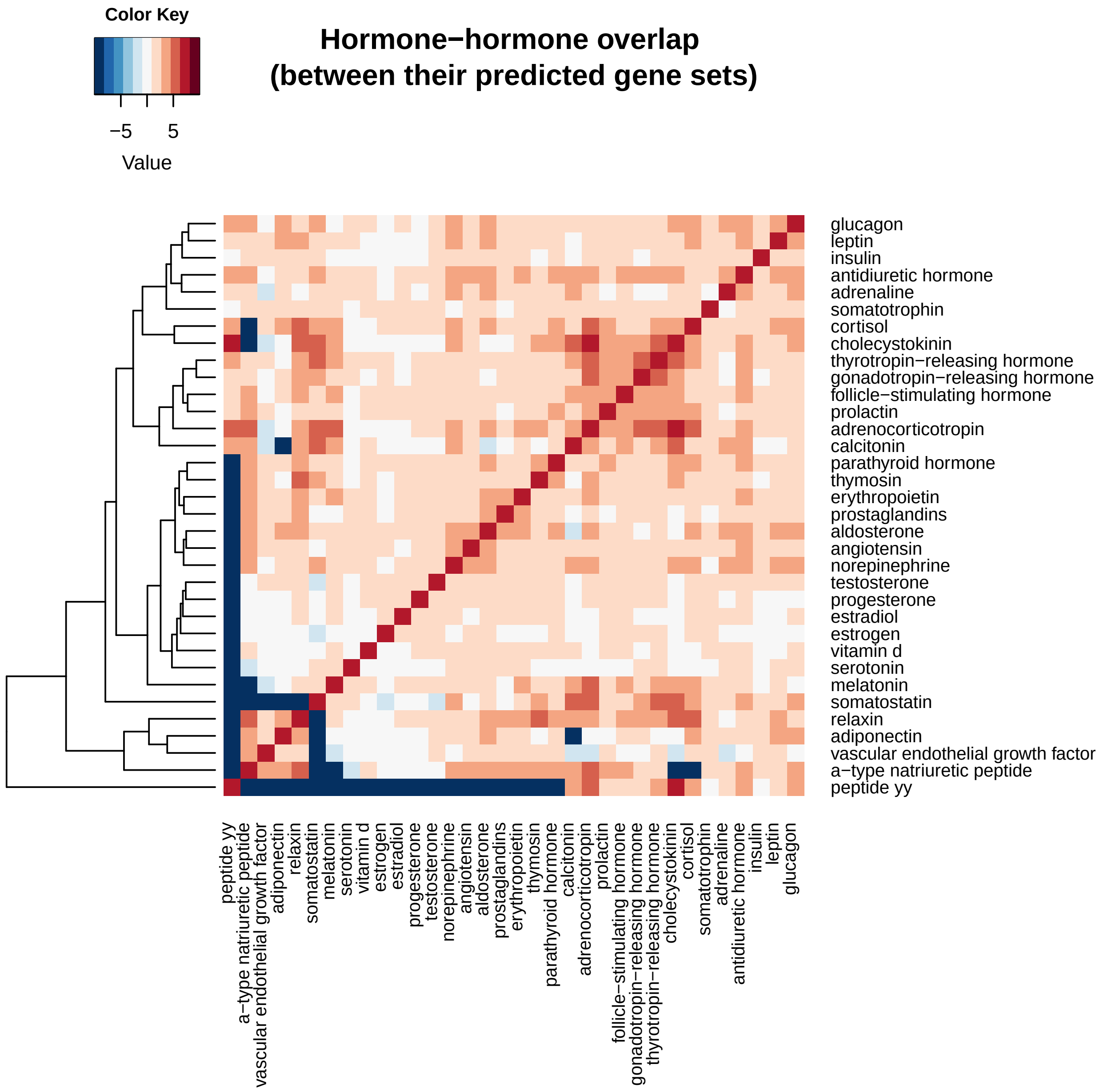

Fig. S3. Pairwise overlap between the highly significant gene predictions of hormones: Let G_i be the set of protein-coding genes predicted for hormone i by BioEmbedS at a high SVM probability score of at least 0.9. The similarity S_{ij} between two hormones i, j is then calculated as the actual overlap between their predicted gene sets, normalized by the overlap expected due to random chance. That is, $S_{ij} = \frac{|G_i \cap G_j|}{(|G_i| \times |G_j|)/N}$, where $N = 19,318$ is the number of all human protein-coding genes considered in this study.

To better visualize and cluster these similarities, this heatmap plots $\log_2(S_{ij} + 0.001)$, with all diagonal entries fixed at the average of the original log-transformed diagonal values. The dendrogram is built using a complete-linkage hierarchical clustering method based on the Euclidean distance metric, as implemented in the R *gplots* package's *heatmap.2* function. Biologically-related hormones like insulin and glucagon are indeed grouped closer together in this dendrogram due to their relatively high similarity.

4 Supplementary Files

Supplementary data/result files listed below are available at this link: https://drive.google.com/drive/folders/1dJI9E9qzr6WWr7AOQ-AIc6elwHJAa5FV?usp=sharing .

Suppl File D1: Hormone-wise performance of BioEmbedS model (averaged across the 5 CV test folds).

Suppl File D2a: Disease enrichment analysis of predicted genes for the (34) primary hormones in the HGv1 dataset.

Suppl File D2b: Disease enrichment analysis of predicted genes for the (17) unseen/external hormones in the HGv1 dataset.
